# Supplementary material for: STAT1 signaling shields T cells from NK cell-mediated cytotoxicity
Source: Nat Commun. 2019 Feb 22;10:912. doi: 10.1038/s41467-019-08743-8 (PMC6385318; doi:10.1038/s41467-019-08743-8)
Supplement: Supplementary file 1 — Supplementary Information [file 41467_2019_8743_MOESM1_ESM.pdf]

STAT1 signaling shields T cells from NK cell mediated cytotoxicity

Kang et. al.

Supplementary Figures

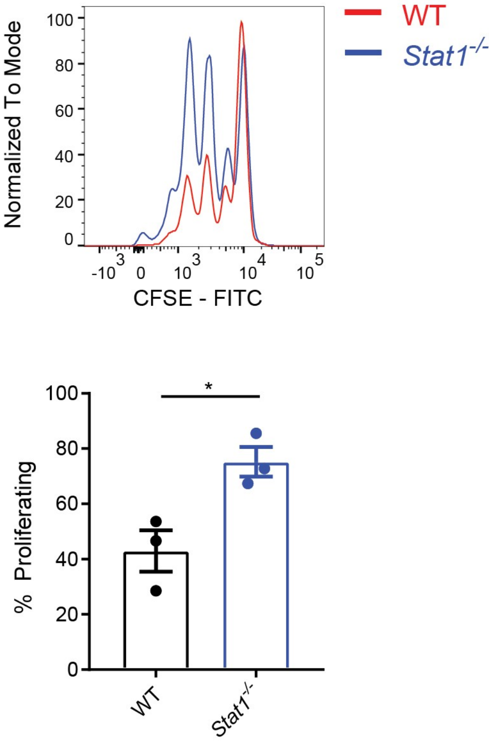

Supplementary Fig. 1 *Stat1$^{-/-}$* T cells do not display defective expansion *in vitro*. WT or *Stat1$^{-/-}$* unfractionated CD4$^+$ T cells were stimulated *in vitro* with anti-CD3 (3μg/ml) and anti-CD28 (1μg/ml) for 3 days and cell proliferation analyzed. Representative flow cytometry plots are shown (gated on live CD4$^+$ cells) with mean frequencies ± SEM. Data are pooled from 3 independent experiments, with each point representing the average across technical replicates in each experiment. *$p<0.05$ by unpaired two-tailed t-test. Refers to Fig. 4.

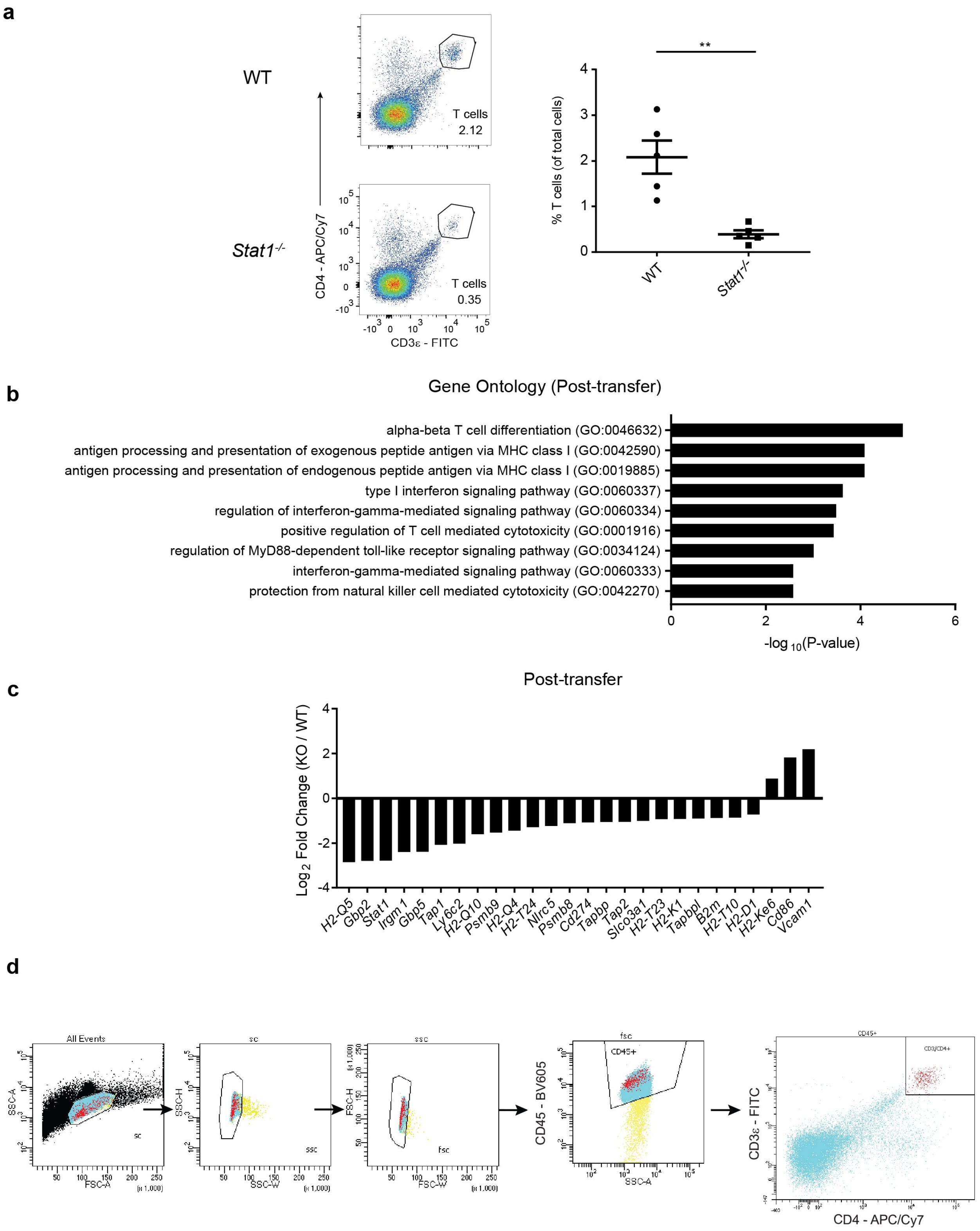

a
WT
Stat1-/-
T cells
2.12
T cells
0.35
CD4 - APC/Cy7
CD3ε - FITC
**
% T cells (of total cells)
WT
Stat1-/-
b
Gene Ontology (Post-transfer)
alpha-beta T cell differentiation (GO:0046632)
antigen processing and presentation of exogenous peptide antigen via MHC class I (GO:0042590)
antigen processing and presentation of endogenous peptide antigen via MHC class I (GO:0019885)
type I interferon signaling pathway (GO:0060337)
regulation of interferon-gamma-mediated signaling pathway (GO:0060334)
positive regulation of T cell mediated cytotoxicity (GO:0001916)
regulation of MyD88-dependent toll-like receptor signaling pathway (GO:0034124)
interferon-gamma-mediated signaling pathway (GO:0060333)
protection from natural killer cell mediated cytotoxicity (GO:0042270)
-log 10(P-value)
c
Post-transfer
Log2 Fold Change (KO / WT)
H2-Q5
Gbp2
Stat1
Irgm1
Gbp5
Tap1
Ly6c2
H2-Q10
Psmb9
H2-Q4
H2-T24
Nlrc5
Psmb8
Cd274
Tapbp
Tap2
Slco3a1
H2-T23
H2-K1
Tapbpl
B2m
H2-T10
H2-D1
H2-Ke6
Cd86
Vcam1
d
All Events
SSC-A
FSC-A
sc
SSC-H
SSC-W
ssc
FSC-H
FSC-W
fsc
CD45+
CD45 - BV605
SSC-A
CD45+
CD3/CD4+
CD3ε - FITC
CD4 - APC/Cy7

Supplementary Fig. 2 Downregulation of the MHC-I pathway in *Stat1*$^{-/-}$ T cells post-transfer. *Rag1*$^{-/-}$ mice were injected i.p. with 5 X 10^6 WT or *Stat1*$^{-/-}$ unfractionated $CD4^+$ T cells and analyzed 1 week post transfer. **(a)** Representative flow cytometry plots of $CD4^+$ T cells (spleen + lymph nodes) post-transfer, along with their mean frequencies ± SEM. Each point represents an individual mouse. **(b)** Selected Gene ontology terms showing differential expression of the MHC class I pathway in *Stat1*$^{-/-}$ T cells post-transfer. **(c)** Similar downregulation of various genes involved in MHC class I antigen presentation in *Stat1*$^{-/-}$ T cells compared to WT T cells in the post-transfer setting. **(d)** Gating strategy for sorting T cells for RNA-seq post transfer. **$p<0.01$ by two-tailed Mann-Whitney test. Refers to Fig. 5.

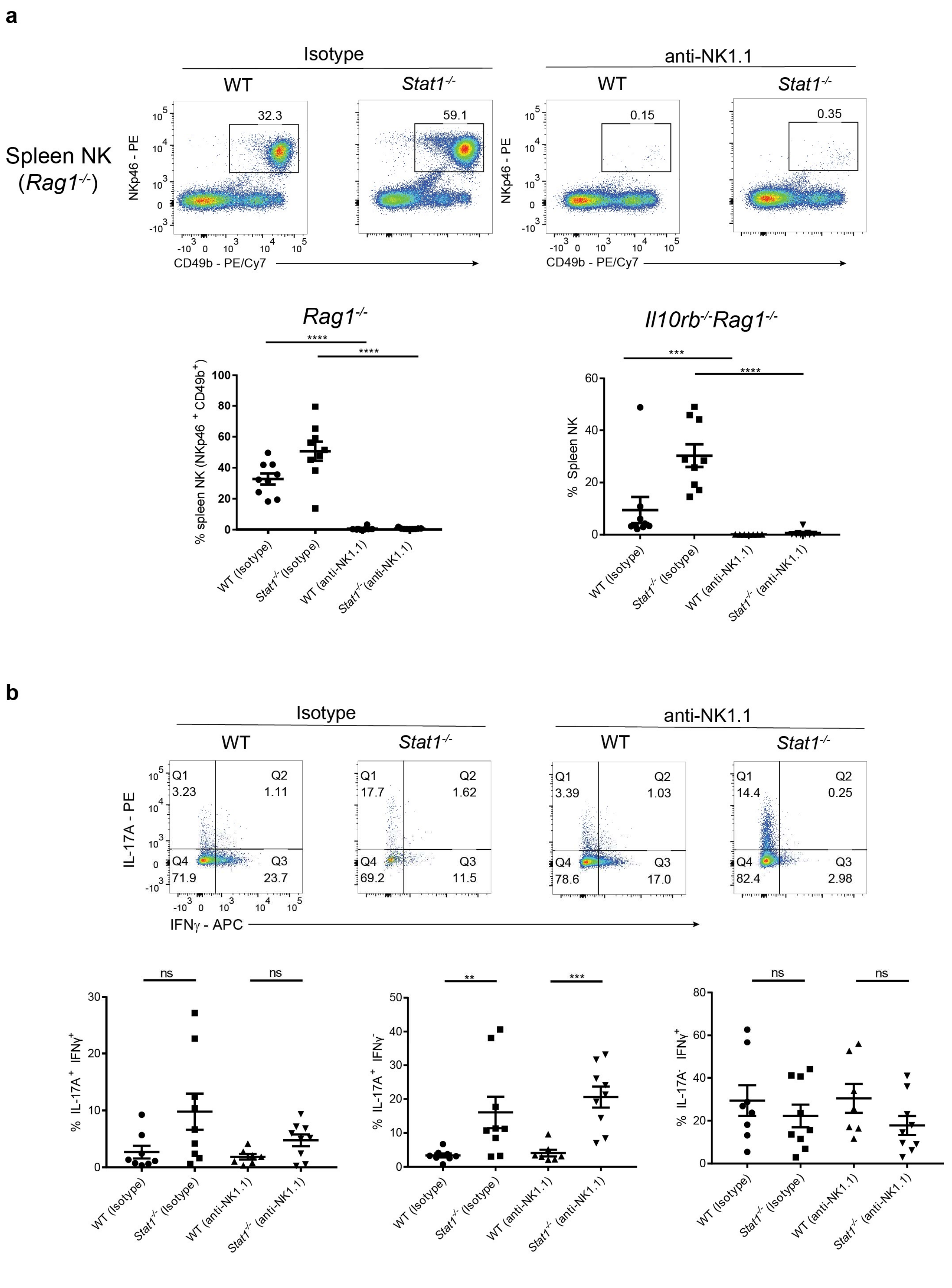
a
Isotype
anti-NK1.1
WT
Stat1-/-
Spleen NK
(Rag1-/-)
NKp46 - PE
CD49b - PE/Cy7
32.3
59.1
0.15
0.35
Rag1-/-
Il10rb-/-Rag1-/-
% spleen NK (NKp46+ CD49b+)
% Spleen NK
****
***
WT (Isotype)
Stat1-/- (Isotype)
WT (anti-NK1.1)
Stat1-/- (anti-NK1.1)
b
Isotype
anti-NK1.1
WT
Stat1-/-
IL-17A - PE
IFNγ - APC
Q1 3.23
Q2 1.11
Q4 71.9
Q3 23.7
Q1 17.7
Q2 1.62
Q4 69.2
Q3 11.5
Q1 3.39
Q2 1.03
Q4 78.6
Q3 17.0
Q1 14.4
Q2 0.25
Q4 82.4
Q3 2.98
ns
**
***
% IL-17A+ IFNγ+
% IL-17A+ IFNγ-
% IL-17A- IFNγ+
WT (Isotype)
Stat1-/- (Isotype)
WT (anti-NK1.1)
Stat1-/- (anti-NK1.1)

Supplementary Fig. 3 NK cell depletion does not alter *Stat1*$^{-/-}$ T cell differentiation *in vivo*. **(a)** anti-NK1.1 depleting antibody effectively removes NK cells. Representative flow cytometry plots of splenic NK cells (NKp46^{+} CD49b^{+}) in *Rag1*$^{-/-}$ mice injected with control or NK cell depleting antibody at 3 weeks post transfer, as well as their mean frequencies ± SEM in both *Rag1*$^{-/-}$ and *Il10rb*$^{-/-}$*Rag1*$^{-/-}$ mice. **(b)** Colonic T cells (gated on live CD45^{+} TCRβ^{+} CD4^{+} T cells) from *Il10rb*$^{-/-}$ *Rag1*$^{-/-}$ mice at 3 weeks post transfer were analyzed for their differentiation profile by IL-17A and IFNγ staining. Representative plots are shown along with their mean frequencies ± SEM. Data pooled from 3 independent experiments, with each point representing an individual mouse. **p<0.01, ***p<0.001, ****p<0.0001 by two-tailed Mann-Whitney test. Refers to Fig. 6.

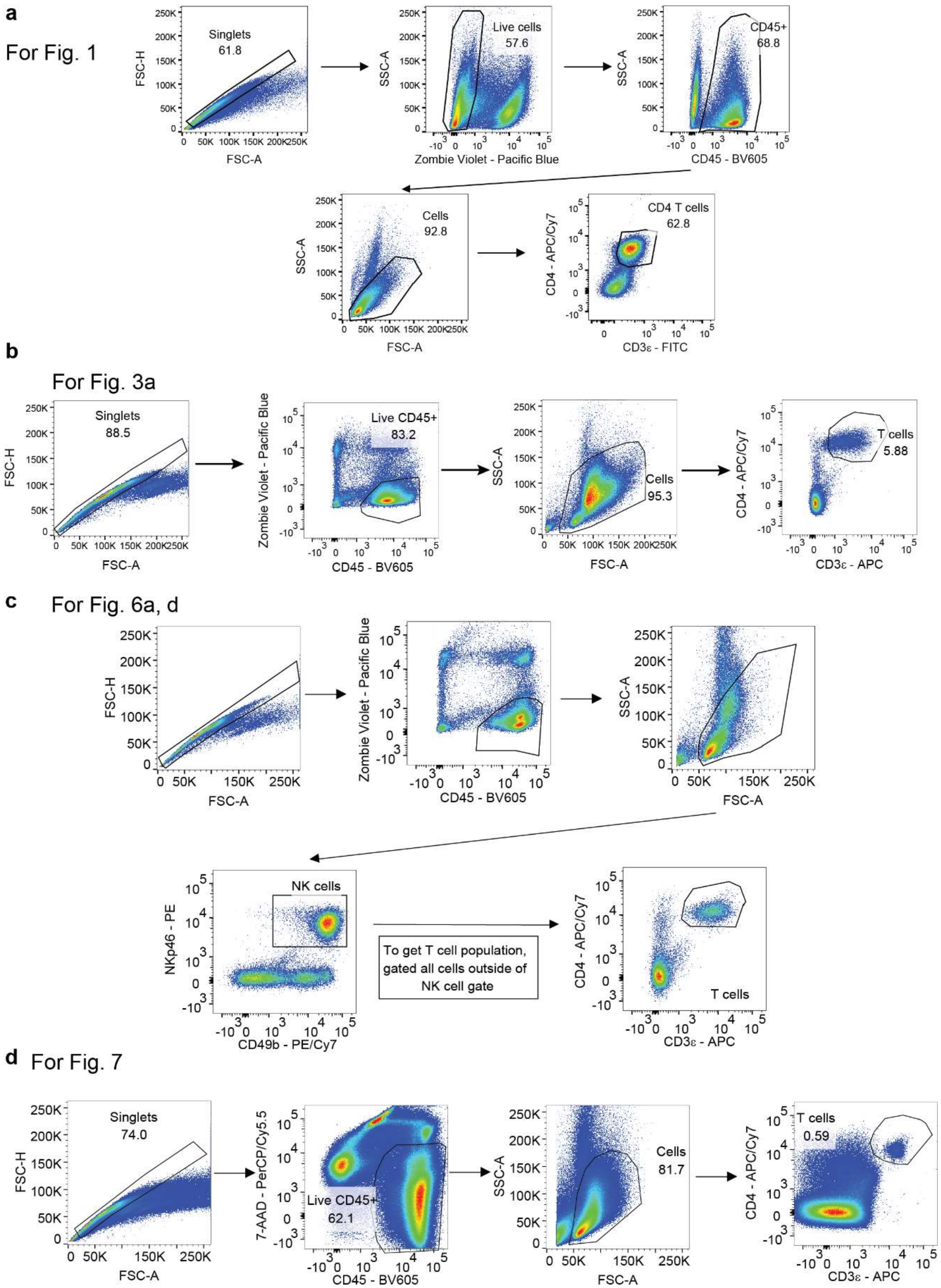

Supplementary Fig. 4 Gating Strategies. Representative gating strategies used for the flow cytometric analyses in **(a)** Fig. 1, **(b)** Fig. 3a, **(c)** Fig. 6a,d and **(d)** Fig. 7.
