# Supplementary material for: STAT1 signaling shields T cells from NK cell-mediated cytotoxicity
Source: Nat Commun. 2019 Feb 22;10:912. doi: 10.1038/s41467-019-08743-8 (PMC6385318; doi:10.1038/s41467-019-08743-8)
Supplement: Supplementary file 3 — Reporting Summary [file 41467_2019_8743_MOESM3_ESM.pdf]

nature research

Corresponding author(s): Scott Snapper

Reporting Summary

Nature Research wishes to improve the reproducibility of the work that we publish. This form provides structure for consistency and transparency in reporting. For further information on Nature Research policies, see Authors & Referees and the Editorial Policy Checklist.

Statistical parameters

When statistical analyses are reported, confirm that the following items are present in the relevant location (e.g. figure legend, table legend, main text, or Methods section).

n/a	Confirmed	
☐	☒	The exact sample size (n) for each experimental group/condition, given as a discrete number and unit of measurement
☐	☒	An indication of whether measurements were taken from distinct samples or whether the same sample was measured repeatedly
☐	☒	The statistical test(s) used AND whether they are one- or two-sided<br>*Only common tests should be described solely by name; describe more complex techniques in the Methods section.*
☐	☒	A description of all covariates tested
☐	☒	A description of any assumptions or corrections, such as tests of normality and adjustment for multiple comparisons
☐	☒	A full description of the statistics including central tendency (e.g. means) or other basic estimates (e.g. regression coefficient) AND variation (e.g. standard deviation) or associated estimates of uncertainty (e.g. confidence intervals)
☐	☒	For null hypothesis testing, the test statistic (e.g. F, t, r) with confidence intervals, effect sizes, degrees of freedom and P value noted<br>*Give P values as exact values whenever suitable.*
☒	☐	For Bayesian analysis, information on the choice of priors and Markov chain Monte Carlo settings
☒	☐	For hierarchical and complex designs, identification of the appropriate level for tests and full reporting of outcomes
☒	☐	Estimates of effect sizes (e.g. Cohen's d, Pearson's r), indicating how they were calculated
☐	☒	Clearly defined error bars<br>*State explicitly what error bars represent (e.g. SD, SE, CI)*

Our web collection on statistics for biologists may be useful.

Software and code

Policy information about availability of computer code

Data collection	For Flow Cytometry, acquisition was performed using FACSDiva.
Data analysis	Flow cytometric data were analyzed using FlowJo v10. Graphs and statistical analyses were performed using Graphpad Prism 7. RNAseq analysis was performed using the VIPER algorithm (Cornwell et. al. 2018 BMC Bioinformatics) which is briefly described in the manuscript (Methods).

For manuscripts utilizing custom algorithms or software that are central to the research but not yet described in published literature, software must be made available to editors/reviewers upon request. We strongly encourage code deposition in a community repository (e.g. GitHub). See the Nature Research guidelines for submitting code & software for further information.

Data

Policy information about availability of data

All manuscripts must include a data availability statement. This statement should provide the following information, where applicable:

- Accession codes, unique identifiers, or web links for publicly available datasets
- A list of figures that have associated raw data
- A description of any restrictions on data availability

All data in this study are available from the corresponding author upon reasonable request. RNAseq data has been deposited in the GEO under GSE116475 and will be made publically available upon acceptance of the manuscript.

Field-specific reporting

Please select the best fit for your research. If you are not sure, read the appropriate sections before making your selection.

☒ Life sciences ☐ Behavioural & social sciences ☐ Ecological, evolutionary & environmental sciences

For a reference copy of the document with all sections, see nature.com/authors/policies/ReportingSummary-flat.pdf

Life sciences study design

All studies must disclose on these points even when the disclosure is negative.

Sample size	For all in vivo experiments, sample sizes were chosen based on our earlier publications. with adequate number of mice being used to achieve statistically significant results based on the Mann-Whitney test or 2-way ANOVA test with Bonferroni's correction. For in vitro experiments, each experiment was replicated at least 3 times to achieve statistically significant results with the 2-tailed t-test.
Data exclusions	For CTV experiments (Fig. 7), T cell analysis was not performed in mice where there were no visible T cells (due to faulty injection). This occurred at random across experimental groups.
Replication	All in vivo experiments were successfully replicated at least 2-3 times and data pooled for the analysis. All in vitro experiments were successfully replicated at least 3 times and pooled for the analysis.
Randomization	For in vivo experiments, experimental groups were randomly assigned such that each group had mice that were controlled for age, sex and housing.
Blinding	Histological scoring of colonic tissue was performed in a blinded fashion. For acquisition of other data, blinding was not performed as it was not necessary.

Reporting for specific materials, systems and methods

Materials & experimental systems

n/a	Involved in the study
☐	☒ Unique biological materials
☐	☒ Antibodies
☒	☐ Eukaryotic cell lines
☒	☐ Palaeontology
☐	☒ Animals and other organisms
☒	☐ Human research participants

Methods

n/a	Involved in the study
☒	☐ ChIP-seq
☐	☒ Flow cytometry
☒	☐ MRI-based neuroimaging

Unique biological materials

Policy information about availability of materials

Obtaining unique materials	All mice are available from commercial sources except for certain strains bred in house that can be made available from the corresponding author upon reasonable request.

Antibodies

Antibodies used	For flow cytometric staining, antibodies against the following were used (Clone name and supplier in brackets): CD3ε (145-2C11, Biolegend), TCRβ (H57-597, Biolegend), CD4 (GK1.5, Biolegend), NKp46 (29A1.4, Biolegend), CD49b (HMα2, Biolegend), CD45 (30-F11, Biolegend), H2-Kb/Db (28-8-6, Biolegend), Qa-2 (695H1-9-9, Biolegend), Qa-1b (6A8.6F10.1A6, Miltenyi Biotec), CD16/32 (93, Biolegend), IL-17A (TC11-18H10.1, Biolegend), IFNγ (XMG1.2, Biolegend/eBioscience), Mouse IgG2a, κ Isotype Ctrl (MOPC-173, Biolegend), Mouse IgG1, κ Isotype Ctrl (MOPC-21, Biolegend), CD45.1 (A20, Biolegend), CD45.2 (104, Biolegend) .<br>For T cell stimulation, functional grade antibodies against CD3ε (145-2C11, eBioscience) and CD28 (37.51, eBioscience) were used.<br>For NK depletion assays, antibodies against NK1.1 (PK136, BioXCell) or Isotype Control (C1.18.4, BioXCell) were used.<br>For macrophage depletion assays, antibodies against CSF1R (AFS98, BioXCell) or Isotype Control (2A3, BioXCell) were used.
Validation	All antibodies for flow cytometry and T cell stimulation were validated by the manufacturer. Antibodies for in vivo NK depletion were validated based on literature and on confirmation of NK cell depletion by flow cytometry.

Animals and other organisms

Policy information about studies involving animals; ARRIVE guidelines recommended for reporting animal research

Laboratory animals	C57BL/6J (Strain 000664), B6.SJL-Ptprca Pepcb/BoyJ (CD45.1, Strain 002014), B6.129S7-Rag1tm1Mom/J (Rag1-/-, Strain 002216), B6.129S(Cg)-Stat1tm1Dlv (Stat1-/-, Strain 012606), B6.Cg-Ifngr1tm1AgtIfnar1tm1.2Ees/J (Ifnar1-/-Ifngr1-/-, Strain 029098) mice were purchased from Jackson Labs. Il10rb-/-Rag1-/- mice on the B6 background were generated by crossing Il10rb-/- mice (a gift from Thaddeus Stappenbeck, Washington University) with Rag1-/- mice. Stat1-/-Rag1-/- mice were generated by crossing Stat1-/- mice with Rag1-/- mice.
Wild animals	The study did not involve wild animals.
Field-collected samples	The study did not involve samples collected from the fields.

Flow Cytometry

Plots

Confirm that:

☒ The axis labels state the marker and fluorochrome used (e.g. CD4-FITC).

☒ The axis scales are clearly visible. Include numbers along axes only for bottom left plot of group (a 'group' is an analysis of identical markers).

☒ All plots are contour plots with outliers or pseudocolor plots.

☒ A numerical value for number of cells or percentage (with statistics) is provided.

Methodology

Sample preparation	Spleen and lymph nodes were physically mashed through a cell strainer. They were then lysed with ACK lysing buffer before analysis.<br>Preparation of colonic lamina propria is described in the manuscript (Methods). Briefly, the large intestine (cecum + colon) was removed of stool content, cut longitudinally and then into transverse sections. These sections were first digested in a buffer containing EDTA, followed by secondary digestion in a buffer containing Collagenase VIII.
Instrument	All data were acquired with a BD FACSCanto II or LSRFortessa cytometer. FACS sorting was performed on a BD FACSAria II.
Software	All flow cytometry data were analyzed with FlowJo v10.
Cell population abundance	Described in the manuscript (Methods)
Gating strategy	Described in the manuscript (Supplementary Figures 2d and 4)

☒ Tick this box to confirm that a figure exemplifying the gating strategy is provided in the Supplementary Information.
